# Comparison of fascia iliaca block with quadratus lumborum block for hip arthroplasty: A meta-analysis of randomized controlled trials

**DOI:** 10.1097/MD.0000000000038247

**Published:** 2024-05-17

**Authors:** Yunqing Guo, Xiaojing Xia, Jialin Deng

**Affiliations:** a Operating Room of Chongqing University Jiangjin Hospital, Chongqing, China; b Orthopedics Department of Chongqing University Jiangjin Hospital, Chongqing, China; c Nursing Department of Chongqing University Jiangjin Hospital, Chongqing, China.

**Keywords:** fascia iliaca block, hip arthroscopy, pain management, quadratus lumborum block, randomized controlled trials

## Abstract

**Background:**

The efficacy of fascia iliaca block (FIB) versus quadratus lumborum block (QLB) remains controversial for pain management of hip arthroplasty. We conduct a systematic review and meta-analysis to explore the influence of FIB versus QLB on the postoperative pain intensity of hip arthroplasty.

**Methods:**

We have searched PubMed, EMbase, Web of Science, EBSCO, and Cochrane Library databases through July 2023 for randomized controlled trials assessing the effect of FIB versus QLB on pain control of hip arthroplasty. This meta-analysis is performed using the random-effect model or fixed-effect model based on the heterogeneity.

**Results:**

Four randomized controlled trials and 234 patients were included in the meta-analysis. Overall, compared with QLB for hip arthroscopy, FIB was associated with substantially lower pain scores at 2 hours (mean difference [MD] = –0.49; 95% CI = –0.63 to –0.35; *P* < .00001) and pain scores at 12 hours (MD = –0.81; 95% CI = –1.36 to –0.26; *P* = .004), but showed no impact on pain scores at 24 hours (MD = –0.21; 95% CI = –0.57 to 0.15; *P* = .25), time to first rescue analgesia (standard mean difference = 0.70; 95% CI = –0.59 to 1.99; *P* = .29), analgesic consumption (MD = –4.80; 95% CI = –16.57 to 6.97; *P* = .42), or nausea and vomiting (odd ratio = 0.66; 95% CI = 0.32–1.35; *P* = .25).

**Conclusions:**

FIB may be better than QLB for pain control after hip arthroplasty, as evidenced by the lower pain scores at 2 and 24 hours.

## 1. Introduction

Many patients suffer from obvious pain after hip arthroplasty and increasing interest is focused on its perioperative analgesia in order to promote early postoperative mobilization and discharge.^[1–3]^ Perioperative pain control is of utmost importance for patients’ recovery and satisfaction.^[4,5]^ Perioperative analgesia is able to reduce opioid consumption, improve immediate postoperative function, and patient satisfaction.^[6–8]^ Regional analgesia techniques have been developed to optimize multimodal analgesia technique in order to improve pain relief, decrease opioid requirements, time to first mobilization, and hospital length of stay.^[9–11]^

Fascia iliaca block (FIB) shows important potential in pain control and the decrease in opioid consumption for surgery. It is easy and fast to be performed.^[12,13]^ FIB has become an increasingly significant option for hip arthroplasty.^[14]^ In addition, as a newer group of blocks, quadratus lumborum blocks (QLB) is conducted by injecting anesthetic through either anterolateral, posterior, or anterior/transmuscular relative to the quadratus lumborum muscle. Previous studies demonstrated the capability of QLB for postoperative pain relief of hip arthroplasty, as evidenced by the decrease in pain scores and opioid use.^[15]^

Several randomized controlled trials (RCTs) compared the analgesic efficacy of FIB with QLB for hip arthroplasty, but the results were not well established.^[16–18]^ With accumulating evidence, this meta-analysis of RCTs aimed to explore the efficacy of FIB versus QLB for pain control after hip arthroplasty. We hypothesized that FIB may have better analgesic profile for hip arthroplasty than QLB due to its easier and faster properties.

## 2. Materials and methods

Ethical approval and patient consent were not required because this was a systematic review and meta-analysis of previously published studies. The systematic review and meta-analysis were conducted and reported in adherence to Preferred Reporting Items for Systematic Reviews and Meta-Analyses.^[19–21]^

### 2.1. Search strategy and study selection

Two investigators independently searched the following databases (inception to July 2023): PubMed, EMbase, Web of Science, EBSCO, and Cochrane Library databases. The electronic search strategy was conducted using the following keywords: “quadratus lumborum block” AND “fascia iliaca block” AND “hip arthroplasty” OR “hip replacement.” We also checked the reference lists of the screened full-text studies to identify other potentially eligible trials. The inclusive selection criteria were as follows: study design was RCT; intervention treatments were FIB versus QLB; patients underwent hip arthroplasty.

### 2.2. Data extraction and outcome measures

We extracted the following information: author, number of patients, age, female, body mass index, American Society of Anesthesiologists Physical Status, detail methods in each group, etc. Data were extracted independently by 2 investigators, and discrepancies were resolved by consensus. The primary outcomes were pain scores at 2 hours and pain scores at 12 hours. Secondary outcomes included pain scores at 24 hours, time to first rescue analgesia, analgesic consumption, as well as nausea and vomiting.

### 2.3. Quality assessment in individual studies

Methodological quality of the included studies was independently evaluated using the modified Jadad Scale.^[22]^ There were 3 items for Jadad Scale: randomization (0–2 points), blinding (0–2 points), dropouts and withdrawals (0–1 points). The score of Jadad Scale varied from 0 to 5 points. An article with Jadad score ≤2 was considered to be of low quality. If the Jadad score ≥3, the study was thought to be of high quality.^[23]^

### 2.4. Statistical analysis

We estimated the standard mean difference or mean difference (MD) with 95% confidence interval (CI) for continuous outcomes and odd ratio with 95% CIs for dichotomous outcomes.^[20,21]^ Heterogeneity was reported using the *I*^2^ statistic, and *I*^2^ > 50% indicated significant heterogeneity.^[24]^ The random-effect model was used for significant heterogeneity, and otherwise the fixed-effect model was applied. Whenever significant heterogeneity was present, we search for potential sources of heterogeneity via omitting 1 study in turn for the meta-analysis or performing subgroup analysis. All statistical analyses were performed using Review Manager Version 5.3 (The Cochrane Collaboration, Software Update, Oxford, UK).

## 3. Results

### 3.1. Literature search, study characteristics, and quality assessment

**Figure 1.**
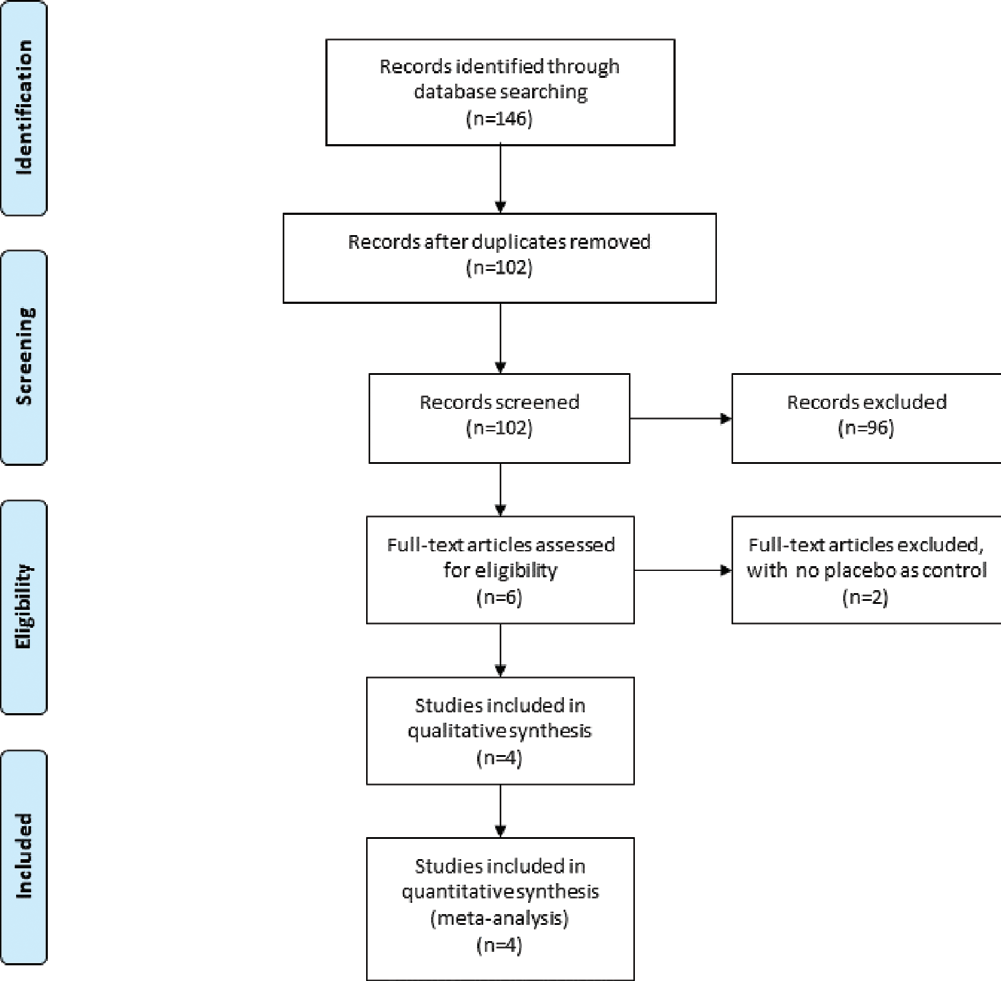
Flow diagram of study searching and selection process.

A detailed flowchart of the search and selection results was shown in Figure 1. Initially, 146 potentially relevant articles were identified. Finally, 4 RCTs that met our inclusion criteria were included in the meta-analysis.^[16–18,25]^ Table 1 demonstrated the baseline characteristics of 4 eligible RCTs in the meta-analysis. The 4 studies were published between 2021 and 2022, and total sample size was 234. QLB and FIB were conducted with bupivacaine or ropivacaine.

**Table 1:** Characteristics of included studies.

No.	Author	FIB group						QLB group						Jadad scores	Patient samples
		Number	Age (yr)	Female (n)	Body mass index (kg/m^2^)	American Society of Anesthesiologists Physical Status (I/II/III)	Methods	Number	Age (yr)	Female (n)	Body mass index (kg/m^2^)	American Society of Anesthesiologists Physical Status (I/II/III)	Methods		
1	Wang 2022^[16]^	50	54.7 ± 12.2	21	24.0 ± 2.5	3/38/9	30 mL of ropivacaine 0.33% with epinephrine 2 µg/mL through FIB	50	53.2 ± 13.4	23	24.3 ± 2.1	5/33/12	30 mL of ropivacaine 0.33% with epinephrine 2 µg/mL through QLB	5	Primary unilateral total hip arthroplasty
2	Hashmi 2022^[17]^	24	–	7	29.14 ± 4.12	3/16/5	FIB with 20 mL of 0.25% bupivacaine	24	–	13	26.81 ± 1.09	4/17/3	Transmuscular QLB with 20 mL 0.25% bupivacaine	4	Elective total hip replacement
3	Xia 2021^[18]^	26	69.88 ± 2.76	15	22.31 ± 3.38	0/12/14	FIB with 20 mL of 0.375% ropivacaine	24	70.88 ± 3.70	14	23.52 ± 2.73	0/11/13	QLB with 20 mL of 0.375% ropivacaine	4	Primary unilateral total hip arthroplasty
4	Nassar 2021^[25]^	19	47 ± 17.6	7	–	–	FIB using 30 mL of 0.25% bupivacaine	17	54 ± 16	9	–	–	Transmuscular QLB using 30 mL of 0.25% bupivacaine	3	Hip replacement surgery

FIB = fascia iliaca block, QLB = quadratus lumborum block.

Among the 4 studies included here, 2 studies reported pain scores at 2 hours and pain scores at 12 hours,^[18,25]^ 3 studies reported pain scores at 24 hours,^[17,18,25]^ 2 studies reported time to first rescue analgesia,^[16,18]^ 3 studies reported analgesic consumption,^[16–18]^ as well as 3 studies reported nausea and vomiting.^[16,18]^ Jadad scores of the 4 included studies varied from 3 to 5, and all studies were considered to have high quality according to quality assessment.

### 3.2. Primary outcomes: pain scores at 2 hours, pain scores at 12 hours

**Figure 2.**
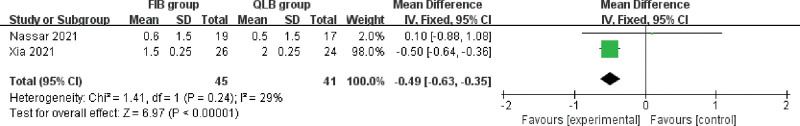
Forest plot for the meta-analysis of pain scores at 2 hours.

**Figure 3.**
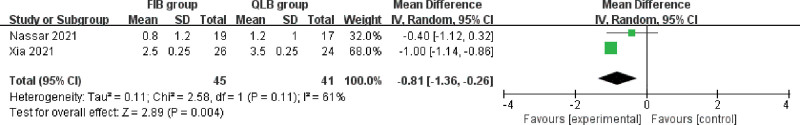
Forest plot for the meta-analysis of pain scores at 12 hours.

Compared to QLB for hip arthroscopy, FIB resulted in significantly reduced pain scores at 2 hours (MD = –0.49; 95% CI = –0.63 to –0.35; *P* < .00001) with low heterogeneity among the studies (*I*^2^ = 29%, heterogeneity *P* = .24, Fig. 2) and pain scores at 12 hours (MD = –0.81; 95% CI = –1.36 to –0.26; *P* = .004) with significant heterogeneity among the studies (*I*^2^ = 61%, heterogeneity *P* = .11, Fig. 3).

### 3.3. Sensitivity analysis

Significant heterogeneity was observed among the included studies for the pain scores at 12 hours. However, we did not perform the sensitivity analysis by omitting 1 study in turn because there were only 2 RCTs.

### 3.4. Secondary outcomes

**Figure 4.**
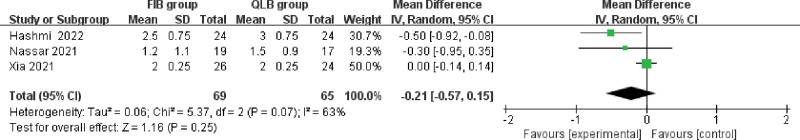
Forest plot for the meta-analysis of pain scores at 24 hours.

**Figure 5.**
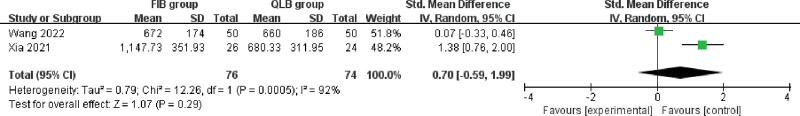
Forest plot for the meta-analysis of time to first rescue analgesia.

**Figure 6.**
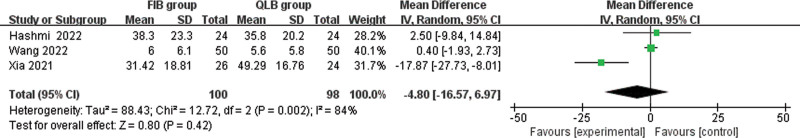
Forest plot for the meta-analysis of analgesic consumption.

**Figure 7.**
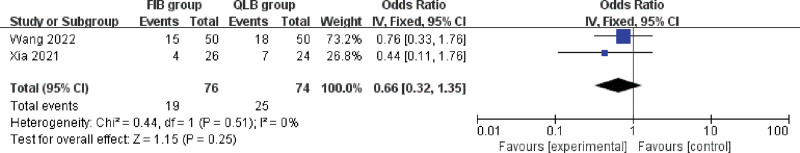
Forest plot for the meta-analysis of nausea and vomiting.

In comparison with QLB for hip arthroplasty, FIB showed no obvious impact on pain scores at 24 hours (MD = –0.21; 95% CI = –0.57 to 0.15; *P* = .25; Fig. 4), time to first rescue analgesia (standard mean difference = 0.70; 95% CI = –0.59 to 1.99; *P* = .29; Fig. 5), analgesic consumption (MD = –4.80; 95% CI = –16.57 to 6.97; *P* = .42; Fig. 6), or nausea and vomiting (odd ratio = 0.66; 95% CI = 0.32–1.35; *P* = .25; Fig. 7).

## 4. Discussion

Many types of peripheral nerve blocks have been developed to minimize perioperative pain and maximize physical function for hip arthroplasty, such as FIB and QLB.^[26,27]^ However, their comparison for pain control is unclear after hip arthroplasty. Our meta-analysis included 4 RCTs and 234 patients undergoing hip arthroplasty. The results revealed that compared to QLB, FIB was able to further reduce pain scores at 2 hours and pain scores at 12 hours, but demonstrated no impact on pain scores at 24 hours, time to first rescue analgesia, analgesic consumption, or nausea and vomiting.

Regarding the sensitivity analysis, significant heterogeneity was seen among the included studies for the pain scores at 12 hours. However, several factors may account for the significant heterogeneity. Firstly, FIB and QLB were conducted with bupivacaine or ropivacaine, which may affect the analgesic assessment. Secondly, the detail methods of FIB and QLB had some difference. For instance, different injection methods were included during QLB, and anterior or transmuscular QLB may have the most consistent upper lumbar plexus spread, which may benefit to improve the analgesic efficacy.^[28]^ Thirdly, these patients all underwent hip arthroplasty, but different operation procedures were needed due to the hip diseases, which may produce various levels of pain intensity.

Narcotic medications is widely used for the traditional pain management for orthopedic surgery, but may result in some side effects on the gastrointestinal, respiratory, integumentary, genitourinary, and neurologic systems.^[29,30]^ Multimodal pain management is extensively developed to improve postoperative pain control and reduce the adverse events. Especially, nerve block such as femoral nerve blocks, FIB, and lumbar plexus blocks showed important potential in improving multimodal pain management. Our meta-analysis aimed to find the ideal nerve block for hip arthroplasty and revealed the better pain relief of FIB than QLB, as shown by the decreased pain scores at 2 hours and pain scores at 12 hours.

This meta-analysis has several potential limitations. Firstly, our analysis is based on 4 RCTs, and more RCTs with large patient samples are needed to confirm our findings. Secondly, there is significant heterogeneity, which may be caused by various kinds and concentrations of analgesics. Thirdly, it is not available to perform the meta-analysis of some important index such as muscle weakness.

## 5. Conclusion

FIB may benefit to improve postoperative pain management than QLB within 12 hours after hip arthroplasty.

## Author contributions

**Conceptualization:** Yunqing Guo, Xiaojing Xia, Jialin Deng.

**Data curation:** Yunqing Guo, Xiaojing Xia, Jialin Deng.

**Formal analysis:** Yunqing Guo, Xiaojing Xia.

**Funding acquisition:** Jialin Deng.

**Investigation:** Yunqing Guo, Xiaojing Xia.

**Methodology:** Yunqing Guo, Xiaojing Xia.

**Project administration:** Yunqing Guo, Xiaojing Xia, Jialin Deng.

**Resources:** Yunqing Guo, Xiaojing Xia, Jialin Deng.

**Software:** Yunqing Guo, Xiaojing Xia, Jialin Deng.

**Supervision:** Yunqing Guo, Jialin Deng.

**Validation:** Jialin Deng.

**Visualization:** Jialin Deng.

**Writing – original draft:** Jialin Deng.

**Writing – review & editing:** Jialin Deng.
